# How and why funders support engaged research

**DOI:** 10.1073/pnas.2400931121

**Published:** 2024-12-30

**Authors:** Angela T. Bednarek, Ben Miyamoto, Kristin Corbett, Charlotte Hudson, Gayle Scarrow, Maeghan Brass, Kim DuMont, Bev Holmes, Lauren Supplee, Robert McLean, Jaspal Bhatia, Shamira Chappell, Maggie Gorman Vélez, Chhaya Kolavalli

**Affiliations:** ^a^The Pew Charitable Trusts, Washington, DC 20004; ^b^Policy and Evaluation Division, International Development Research Centre, Ottawa, ON K1G 3H9, Canada; ^c^Lenfest Ocean Program, The Pew Charitable Trusts, Washington, DC 20004; ^d^Michael Smith Health Research BC, Vancouver, BC V6H 3X8, Canada; ^e^Office for Coastal Management, University of Michigan Water Center, Host of the National Estuarine Research Reserve System (NERRS) Science Collaborative through a Cooperative Agreement with National Oceanic and Atmospheric Administration (NOAA), Ann Arbor, MI 48104; ^f^The William T. Grant Foundation, New York, NY 10165; ^g^United States Department of Health and Human Services, Administration for Children and Families, Office of Planning, Research and Evaluation, Washington, DC 20201; ^h^American Institutes for Research, Arlington, VA 22202; ^i^The Donaghue Foundation, West Hartford, CT 06107; ^j^Ewing Marion Kauffman Foundation, Kansas City, MO 64110

**Keywords:** philanthropy, research-practice partnerships, community engaged research

## Abstract

Research that better aligns policy, practice, and research communities is gaining momentum around the world. This includes engaged research strategies that bring partners, and their diverse perspectives and kinds of knowledge, together to shape research agendas with on-the-ground-needs and to create dynamic problem-solving processes. These approaches aim to generate more equitable and effective solutions to societal challenges. Although many of these partnered strategies have a longstanding history, entrenched research cultures, practices, and institutional structures stand in the way of scaling them. Given the outsized role funders play in shaping research efforts, funders are a critical lever for change. This perspective describes the efforts of a global collaborative of philanthropic and public funders who are adapting their practices, supporting the development of infrastructure (e.g., capacity-strengthening, facilitation expertise, processes to guide relational work, etc.), and targeting system-level challenges to enable engaged research to maximize its potential. The authors integrate insights from different issue areas, geographies, and funding areas to provide concrete examples of funder activities that support engaged research and to suggest areas for further action. Recommendations include scaling changes in funding practices, deepening understanding of how and when engaged research leads to improved outcomes, and reshaping how success is defined and measured.

Partnerships between researchers and those who are affected by or addressing complex societal problems are being piloted and expanded around the world (e.g., refs. 1–9). In Bangladesh, for example, researchers are collaborating directly with urban poor working women and decision-makers to co-design policies and test interventions to prevent future infectious disease outbreaks and pandemics (10). In the United States and elsewhere, researchers are collaborating with coastal communities and decision makers to co-develop and apply knowledge to address critical coastal management issues in the midst of a rapidly changing climate (11, 12). Patients, health practitioners, and health policy makers are partnering with researchers to address critical questions about how to improve healthcare delivery for older adults in rural and remote communities across British Columbia, Canada (13, 14).

These are just a few examples of what we call engaged research in this perspective. These collaborative approaches aim to “[generate] knowledge where researchers work in partnership with research beneficiaries and/or other research users. Together, [these partners] aim to identify problems worth solving, design a research strategy that makes sense to all involved, interpret the meaning and merit of what is discovered for each party, and share and possibly implement findings collaboratively” (9).

Even as these strategies garner interest, there is insufficient infrastructure for undertaking engaged research (3, 15–18). We define infrastructure here as capacity and skill-building that helps partners to engage with each other, expert facilitation for relationship-building, best practices to guide efforts, supportive contexts in which to undertake engaged research, and sufficient funding for its unique needs, among other elements (15–20). Although there are some fundamental tenets of engaged research (e.g., meaningful relationship-building, integrating diverse perspectives and knowledge), much of the infrastructure is underdeveloped, as is the understanding of how to consistently operationalize these principles (3, 15–20).

This perspective shares the learning that a global, cross-sectoral group of funders have surfaced as they have worked to support engaged research—both through their own individual practices and through efforts to learn and act together as peers. We, the authors, describe our motivations and known barriers, share our efforts to adapt funding practices to support these approaches, and suggest areas for further reflection and action, including the need for more empirical studies of engaged research to allow us to invest time and resources wisely. We recognize that engaged research is one of many ways to develop evidence-informed, equitable, and durable solutions for societal challenges. Thus, it is incumbent on us, as funders, to consider carefully when it makes sense to support engaged research, the conditions that allow it to improve outcomes, and the guardrails needed to minimize unintended consequences (3, 21). We intend these details to be useful both to those funders considering supporting engaged research and to those already supporting it but are seeking to improve their practices or build the enabling conditions and infrastructure for it.

## Interest in Engaged Research

Engaged research goes by many names, including “research-practice partnerships” (22), “community-engaged research” (23), “co-production” (24), “transdisciplinary” research (25), “collaborative science” (e.g., ref. 26), and “integrated knowledge translation” (27). Models for this kind of work are not new. Long-standing engaged research methodologies and related approaches exist in many fields and knowledge systems (28–33).

Scholarship suggests an array of potential benefits (2–8, 32–34). For example, studies of engaged research describe how these collaborations can increase the use of research in decision-making about educational curricula (e.g., ref. 5), help achieve sustainability solutions (e.g., refs. 3, and 6), and guide public health policy improvements (e.g., ref. 35). Studies point to key elements of effectiveness, including mutual problem definition between partners to ensure research is relevant for on-the-ground needs, relationship building between participants to generate a sense of legitimacy and trust in the process and outputs, and sufficient time for collaborative sense-making and interpretation of research findings (5, 19, 36–38).

There are also indications that making the research process more inclusive—by including a wide range of perspectives and types of knowledge, such as lived experience (39) and Indigenous knowledge (40)—may increase the chances that resulting solutions are more innovative, useful, and equitable (40–44). For example, a recent review of climate adaptation efforts identified the importance of involving users as partners in research processes as a way to learn from those who are closest to the impacts of climate change. Indeed, “[t]he more culturally sensitive these research processes are, the more successful they can be in incorporating nuanced knowledge that can translate into mutually owned and culturally relevant findings and recommendations…[allowing] the solutions to be embraced by local communities and groups” (44). Finally, engaged research can also complement and integrate with other kinds of research strategies (e.g., basic, discovery), in addition to other types of knowledge (lived experience), to help strengthen fundamental understanding of a particular problem and real-world usefulness (45).

## Support Structures Needed for Engaged Research

These potential benefits drive our interest in supporting engaged research approaches. Yet, these strategies are not a panacea and come with challenges and costs that require attention to local histories, professional roles, and relationship dynamics (46–50). For example, collaborations may result in partnership fatigue, interpersonal tensions, strains on available resources, and exacerbation of unequal power dynamics (3, 21, 50, 51).

These challenges can compound if engaged research efforts are implemented poorly and without sufficient infrastructure, including partner capacities and best practices. Researchers may lack the time or training to participate fully in engaged research efforts (52) or may find engagement conflicts with academic identity and autonomy (e.g., ref. 53). Likewise, partners within policy, practice, and community settings may require support and resources to enable their involvement (54). For example, policymakers, practitioners, and other groups outside academia may find it difficult to take the time to participate (21, 50). Even if partners can collaborate, there are relational challenges that can impede or derail partnerships, ranging from essential language and cultural differences to uncertainty about how to communicate outside one’s professional circles or fears that potential collaborators may not respect one’s time, expertise, or constraints (55). Dedicated facilitation capacity for engaged research (e.g., via boundary spanners or knowledge brokers) can be deployed to manage relationships and ensure effective communication between partners (16, 52, 56, 57), but these skillsets and capacities are not yet mainstreamed (56).

Evaluating engaged research can be challenging. Traditional scientific metrics (e.g., peer-reviewed publications and citation counts) do not capture important outcomes of engaged research, such as relationship development, changes in how partners view a problem or the feasibility of solutions (58–64). Similarly, more work is needed to build understanding about whether, how, and why these engaged research methodologies contribute to improved societal outcomes (3, 15, 16, 21, 65–68).

In this perspective, we share our individual and collective experiences working to support and scale engaged research. We are intent on identifying the key supports required to enable and incentivize engaged research, while paying close attention to understanding whether this approach results in the outcomes we strive to achieve. We acknowledge the decision to support engaged research requires careful, contextual consideration and offer the examples and reflections below as a starting point.

## The Role of Funders in Supporting Engaged Research

Funders can play a critical role in supporting engaged research. Targeted funding calls can incentivize engaged research by providing direct financial support, especially if the calls are widely accessible or involve high-profile funding agencies with significant resources (69). Funders can also build infrastructure for engaged research by adapting their funding practices (e.g., developing funding priorities with research users) (70), or directly supporting infrastructure needs (e.g., grants to develop evaluative measures or supportive processes within institutions) (71, 72). They can also build best practices by supporting empirical studies about how engaged research works and contributes to intended outcomes (16, 73–75).

A collaborative of research funders from around the world, including the authors of this paper, have been exploring these levers for enabling engaged research. The Transforming Evidence Funders Network (TEFN), a group of philanthropies and public funders working across a wide range of issue areas (e.g., public health, education, entrepreneurship, environment) and geographies, is identifying ways to support engaged research (as well as other strategies) with the aim of closing the gap between research and better and more equitable outcomes (76). These funders are investing in engaged research, adapting their funding practices to better support it, and leveraging their resources and influence to address cross-cutting challenges (e.g., academic reward systems) (16, 73–75).

## Funding Practices to Support Engaged Research

To identify practices that participants in TEFN are using to support engaged research, we developed a framework that included key phases of the funding cycle largely based on a rubric created by Arnott et al. (77). These categories include: 1) identifying funding priorities; 2) setting expectations through funding criteria; 3) assessing proposals; 4) supporting implementation; and 5) evaluating process and impact. Funders participating in TEFN have deployed supportive practices in many of these categories. The differentiation between categories is simply intended to facilitate discussion; many of the practices described could fit in several categories. We describe the examples below as field-tested, promising practices because there are too few empirical studies to delineate best practices (78–80).

The examples presented are not a comprehensive picture of promising practices, nor do they signal that funders active in TEFN are the only ones supporting engaged research. Indeed, many communities around the world have long histories bridging different ways of knowing (28–31, 33). Rather we offer ideas and examples humbly, appreciating that our contributions build on the experience of others. With that said, TEFN is a relatively large sample size of research funders (more than 80 distinct funding organizations around the world, with more than 35 engaged specifically in sharing funding practices), so the examples and reflections shared offer a reasonable starting place for identifying promising practices and areas for further analysis and action. The practices below were selected to represent a range of activities happening across the funding life cycle. Activities were deemed to be promising by self-assessment from funders who have implemented them and by their alignment with the related literature about engaged research, where it exists. As we continue to work together in this funder group and with others, including scholars of engaged research, we hope to build on the literature and funders’ tacit knowledge to map out best practices, identify practice trends across the funding community, and increase knowledge about the scalability of these practices. For now, these aspirations are beyond the scope of this paper.

## Identifying Funding Priorities

TEFN participants have found that support for engaged research needs to be intentional from the start. This includes shaping funding priorities to reflect on-the-ground needs. To do so, funders use a variety of mechanisms to ensure potential partners, including community organizations, front-line service organizations, and decision-makers inform those priorities. This process can help lay the groundwork for goal alignment and willingness to partner among those who will be collaborating (70).

Recognizing that potential partners (e.g., users or beneficiaries of research, or other knowledge holders) may not have experience participating in research agenda-setting, funders have worked to find alternative routes to enable them to inform funding priorities. For example, the AIR Equity Initiative at the American Institutes for Research, a self-funded philanthropic initiative that aims to address systemic inequities by race and place in the United States and globally (81), used a multistep process in one of their funding efforts. A wide range of education stakeholders—including youth, communities, researchers, legal and policy professionals, and practitioners—were invited to submit essays on new and reinvigorated approaches to promote school integration (82). After review by a representative reader panel, the AIR Equity Initiative published a selection of essays on their website and several were awarded rapid-cycle project grants to generate and share findings and resources relatively quickly (83). These projects and ideas then informed the scope of the AIR Equity Initiative’s next education grant competition (81).

The Patient-Centered Outcomes Research Institute (PCORI), a patient-centered comparative clinical effectiveness research funder in the United States, uses advisory panels to integrate perspectives from the healthcare community into their research funding priorities. Each of PCORI’s advisory panels must include “representatives of practicing and research clinicians, patients, and experts in scientific and health services research, health services delivery, and evidence-based medicine who have experience in the relevant topic…” (84). The panels provide advice and recommendations related to the refinement of research questions, technical topics, and other questions relevant to PCORI’s work (84).

Other funders conduct regular information needs assessment to identify areas to target for funding (85). The National Oceanic and Atmospheric Administration’s (NOAA) National Estuarine Reserve System (NERRS) Science Collaborative, hosted by the University of Michigan Water Center, provides funding opportunities for communities to address critical coastal management needs in 30 coastal sites across the United States. To ensure the program funds science that serves this large and diverse network, the NERRS Science Collaborative solicits reserve management and cross-cutting science needs annually from every reserve (86).

## Setting Expectations through Funding Criteria

When soliciting proposals, some funders have created rubrics to help set expectations for potential funding recipients and incentivize high-quality researcher collaboration with relevant users and beneficiaries.

For example, the Lenfest Ocean Program, which funds research projects around the world that “address the needs of marine and coastal stakeholders and supports grantees who will engage with the people most likely to use the results,” has developed a framework that specifies expectations of high-quality engaged research grant proposals (12, 85, 87). This framework was made public so it is clear to prospective grantees that the funding program expects successful projects to address a research need identified by a decision-maker, practitioner, rights-holder or community member. The rubric also provides expectations about how researchers will develop research questions with partners and the need for policy and practice-relevant outcomes, beyond scientific publications produced (85).

Funders are also being explicit about expectations for project team composition and provide specific guidance to help teams manage staffing (e.g., including research users from the health system on the project team) (73, 88), allocate funds for relationship-building activities (e.g., funds for travel and meals) (85, 89), and budget for staff time needed to engage (90). For example, Health Research BC, British Columbia, Canada’s health research agency, has team composition requirements and budgetary guidance for engaged research proposals. Teams applying for funding from the grant program, “Convening & Collaborating Competition” must include researcher and research user co-leads and a research or health professional trainee (e.g., student or clinical fellow), which also helps build capabilities within partnering organizations. Health Research BC also provides budgetary guidance that allows for “direct costs associated with bringing people together for the purposes of collaborating, networking and knowledge exchange, including planning, co-ordination, translation, and/or outreach activities” as well supporting salary for participating partners (91).

Similarly, the NERRS Science Collaborative requires that all proposals include a collaborative lead, who is responsible for managing a project process that ensures meaningful user input and responsiveness to that input (80). The program also requires that the project is sufficiently resourced to support equitable and meaningful participation of project partners (e.g., travel support, honoraria, compensation for childcare to attend a meeting, provision of traditional food/gifts that have cultural significance when convening partners).

Recognizing that potential funding recipients may not have existing relationships in place, or may need support for the time and effort required to collaboratively design research agendas, some funders are supporting pilot or planning grants. For example, the U.S. Department of Health and Human Services Agency for Children and Families Office of Planning, Research and Evaluation (OPRE) has used a 2-y planning grant to provide funding recipients the time and resources required to build relationships (88, 90, 92) and develop necessary organizational processes and arrangements (5). OPRE has launched a request for proposals to offer the cohort teams a second grant, enabling them to implement what they developed (80, 92). Other funders, such as Canada’s International Development Research Centre (IDRC) and Global Affairs Canada, are experimenting with shorter two-stage call for proposals processes that afford shortlisted applicants the opportunity to benefit from small grants and additional time to cement engaged research partnerships and co-develop full proposals (93).

Some funders are also paying closer attention to how engaged research teams are managing relationships within their partnership (94, 95). For example, a call for funding proposals from the Climate Adaptation and Resilience Initiative, funded by IDRC and the U.K. Foreign, Commonwealth and Development Office (FCDO), asked applicants to set clear expectations around the roles and responsibilities of the different engaged research partners (96). Teams were encouraged to describe partner expectations, decision-making processes, who is participating, risk management strategies, and protocols about how contributions, authorship, and intellectual property will be allocated and recognized (95).

## Assessing Proposals

Some funders are piloting criteria and processes to improve their ability to assess the quality of engaged research proposals received, including ensuring proposals align with information gaps surfaced by community leaders, policymakers, or other partners, and that they include meaningful engagement (95, 97).

One common route is by reconceptualizing review panels. Some funders are inviting a wider variety of experts to constitute review panels, including individuals most proximate to the issues targeted by the funding call (77). In addition to using review panels for selecting funding themes, PCORI, for example, includes patients and other actors in the healthcare system (e.g., health system administrators, clinicians) as proposal reviewers (98, 99). During review, researchers score five assessment criteria: patient-centeredness, engagement of patients and other stakeholders‚ technical merit, impact of the condition being studied, and likelihood to improve health care and outcomes. Patient and stakeholder reviewers score three criteria: potential to improve care, patient-centeredness, and engagement. Final review includes scientists, patients and other stakeholders before PCORI staff and board finalize funding decisions (99).

In conjunction with broadening review panels, funders are developing rubrics to help applicants understand the expectations associated with the engaged research funding opportunity and help reviewers assess the strength of previous engagements or relationship-building plans within proposals. For example, the Ewing Marion Kauffman Foundation in the United States, which focuses on supporting “financial stability, upward mobility, and economic prosperity—regardless of race, gender, or geography,” piloted a funding proposal scoring rubric for their community-engaged entrepreneurship research funding call that included specific questions focused on engagement processes (43, 100). This included prompting reviewers to rate the extent to which project teams designed mechanisms to iteratively learn from and incorporate the perspectives of people with lived experience in an issue of interest. For example, reviewers were asked to evaluate proposals based on the project team’s ability to engage with the communities who are named in the research and on their plans to create feedback loops that integrate the insights and perspectives of those with lived experience in the research topic (43). In other examples, the Lenfest Ocean Program and the NERRS Science Collaborative both prompt applicants to name intended users and partners, explain how they have been involved in proposal development, and describe the engagement strategy for the full project cycle (85, 101). Their proposal evaluation criteria assess these elements, including intended users’ interest and commitment to sustained involvement in the project, strategies for integrating user or partner input, plans for maintaining relationships between partners, and resources allocated to support the collaborative activities (e.g., budget includes funds for meetings or funds to compensate users for their contributions) (77, 101).

Funders are also developing strategies for assessing and refining these review processes to ensure they are producing intended outcomes. The William T. Grant Foundation, which “invests in high-quality research focused on reducing inequality in youth outcomes and improving the use of research evidence in decisions that affect young people in the United States,” is in the early stage of incorporating youth perspectives into their review process by including youth in the review of letters of inquiry (102). While this new practice rolls out, the foundation’s program teams are also systematically reflecting on their engagement with youth in the review process to draw out insights that can inform the foundation’s operations and even be shared with other funders.

## Supporting Implementation

Some funders provide direct support to help funding recipients conduct engaged research efforts, recognizing that many of these capabilities are still underdeveloped and underresourced (16, 21, 52, 57, 103–106).

For example, the Pacific Institute for Climate Solutions (PICS)—which cultivates partnerships among researchers and “diverse communities (of culture, identity, practice, interest, or place) to co-design climate actions rooted in shared goals and a sense of place” in British Columbia, Canada—provides training to help teams shape their engaged research projects (107). The program has offered “impact pathways” training to grantees to help them identify potential barriers, impact allies (i.e., potential partners), and anticipated outcomes (16, 108). For large, flagship projects, PICS has also hired a full-time staff member to facilitate the engaged research process (16).

The NERRS Science Collaborative dedicates significant support to grantees. Once the project is underway, program staff check in regularly with project teams about the engagement progress. Teams typically share what is working well, where they are experiencing challenges, and/or where they are adjusting to enhance collaboration. Program officers offer reflections and advice where appropriate (80). Grantees also have regular peer learning opportunities via the program’s virtual collaborative science workshop series.

Some funders support consortia or networks of engaged research partners to strengthen and scale capacity more broadly. For example, the Collaborative Adaptation Research Initiative in Africa and Asia supported four consortia that each assembled groups of researchers, think tanks, nongovernmental organizations, and government agencies to identify critical evidence synthesis needs for a variety of contexts (50). With overarching facilitation, leadership, and long-term flexible funding provided across the four consortia, this effort created economies of scale to bring together a wide diversity of partners and a sustained focus on finding common ground.

## Evaluating Process and Impact

Evaluating engaged research requires careful attention to how critical elements of the process are working (e.g., relationship-building), which context-specific factors are influencing the collaboration (e.g., organizational resource constraints, political landscape), how partners are aligning around shared goals, and more (77, 109). To address these needs, funders are supporting the development and application of evaluative frameworks and metrics tailored for engaged research (60, 110).

For example, the William T. Grant Foundation has supported the development of an evaluative framework that details five dimensions of the health of an engaged research effort: 1) building trust and relationships; 2) conducting rigorous research to inform action; 3) supporting practice partners in achieving their own goals; 4) generating broadly relevant knowledge; and 5) building the capacity of both the research organizations and the practice organizations to undertake engagement (58). In later work, the foundation supported the development of a set of indicators for each dimension to help partners use the framework as a self-assessment tool (111). For example, in the survey, there are eight indicators for the dimension of trust that partners use to rate how strongly they agree or disagree with statements about partners: Routinely working together, following through on their commitments, valuing diverse perspectives, navigating conflict in a constructive manner, interrupting problematic power and privilege dynamics, investing in one another’s welfare, navigating broader demands and constraints, and acknowledging context and history (111). Partners use their responses to these indicators to adjust their work together.

Similarly, IDRC and partners, including the Integrated Knowledge Translation Research Network, developed a novel framework for assessing the quality of engaged research. The Research Quality Plus for Co-Production (RQ+ 4 Co-Pro) approach details three tenets: 1) context matters; 2) engaged research quality is multidimensional and should reflect the values and objectives of all partners; and 3) evaluations must draw upon the experience and expertise of all co-producers, not just the researchers. The framework encourages users (who may be scientists or community partners) to focus assessments on three equally important dimensions: 1) rigor (of the research and its engagement approach); 2) legitimacy (including how projects integrate local knowledge and ways of knowing, balance power, build trust, and anticipate negative consequences); and 3) how the research is positioned for real-world use (112). IDRC developed, validated, and refined the framework through field-tests and qualitative interviews with researchers and research-users/beneficiaries (112).

To evaluate their project outcomes, the Lenfest Ocean Program defined impact categories based on their program goal of supporting research to inform policy (63). These categories include the following: contributed to science (e.g., published scientific journal articles or other technical reports), prompted dialog (e.g., research results garnering media attention), informed decision-making, management, or policy (e.g., research results were considered directly in discussions by government agencies or resource managers), and influenced decision-making, management, or policy (e.g., research results directly contributed to a new management measure or policy) (63). The Lenfest team develops impact narratives for projects based on these categories and periodically uses external reviews to track project outcomes and impacts and identify which project elements (e.g., use-driven research questions; sustained engagement between researcher and community partner) were key to the observed outcomes (103).

## Beyond Funding Practices

Our work to identify funding practices that meaningfully support engaged research has revealed that we and other funders still have much to learn. We need to ensure we are effectively supporting engaged research, and better mitigating unintended consequences, such as partnership fatigue and unequal power dynamics (e.g., refs. 21, 40, and 44).

Yet, careful self-reflection on our practices is not sufficient. We need to increase rigorous understanding about whether, how, and to what extent engaged research contributes to improved outcomes. This understanding should include which specific elements of the process are critical to success and adapt our own practices accordingly. We also need to be receptive to the possibility that engaged research will not always be the right strategy.

We also recognize that reshaping funding practices can only take us so far because these efforts, while important, have a limited reach into the broader systems and infrastructure that mediate the interactions among the diverse groups and the collaborations we are trying to support (113). Therefore, we see a need for greater attention to cross-cutting challenges. Below, we share examples of some of the gaps and where we see the need for more aligned and collective action.

## Strengthen Research about Engaged Research and Pathways to Better Outcomes

Several funders have recognized the critical importance of investing in rigorous, empirical studies that test the connections between interventions (e.g., relationship-building) and intended outcomes (e.g., improved and more equitable educational outcomes). For example, the William T. Grant Foundation has a longstanding call for proposals that invites researchers to study what conditions support the use of research evidence in policy making and practice, how to cultivate those conditions, and if and how improved use of research in decision-making leads to better outcomes for children. Early in this initiative, research-practice partnerships (i.e., engaged research) emerged as a promising means of generating findings that are more likely to be trusted and used in decision-making to improve conditions for youth (114).

The William T. Grant Foundation continues to support studies that strengthen knowledge about engaged research, research use, and outcomes and has invested in a methods repository to support this work (115). Yet, while many community and public agency leaders express interest in these findings, relatively few researchers respond to the funding calls and relatively few funders support such research. An exception is the “Science of Engagement” funding call PCORI launched in 2022 to support studies that develop understanding about effective engaged research strategies and build knowledge about whether and how these strategies achieve their targeted outcomes (e.g., reducing health disparities) (116, 117).

Despite these efforts, there is still much to be learned about whether, why, and how engaged research ultimately leads to improved outcomes (118). These questions must be addressed with rigorous studies—to provide sound insights about how to effectively improve engaged research strategies, to ensure good stewardship of partners’ time and resources, and to allow shifts to other strategies if outcomes are not improving. This will require a concerted effort to build on and expand existing scholarship (e.g., studies of engaged research impacts in different social and economic settings) and to ensure this scholarship informs practice (119–121).

## Consider the Ecosystem

Engaged research involves partnerships between very different actors, with different institutional contexts and challenges that can hamper participation. For example, researchers struggle with misaligned academic norms, unfamiliarity with engaged research practices, and insufficient infrastructure and practices for collaboration with partners outside the research system (e.g., intellectual property policies that limit joint control of collaboratively created products) (18, 122, 123). Several TEFN participants have addressed some of these challenges with funding programs targeted at building capacity and incentives within universities to reward and support engaged research (18). The U.S. NSF recently initiated a new large-scale funding program of over $100 million USD dedicated over 4 y for the “Accelerating Research Translation” program (124) to build institutional capacity and infrastructure needed for engaged research conducted in partnership with governments, nonprofits, industry, and others (125). Other funders within TEFN have created grant programs such as the “Institutional Challenge Grant” (William T. Grant Foundation and other funder partners) or “Bridging the Gap” (Carnegie Corporation of New York) to incentivize U.S. universities to undertake engaged research and develop capabilities to reward and assess engaged research, such as how to credit engaged research outcomes as scholarship in promotion and tenure decisions (71, 72).

However, a sole focus on building the capacity of researchers and research institutions is not sufficient (87). Without attention to each partners’ specific needs, partnerships may never get off the ground or get derailed if the partnerships’ relationships or capacities are unbalanced. One promising route to balancing partnerships involves shifting project leadership to a partner not at a conventional research institution. For example, the Community Partnerships to Advance Science for Society program at the U.S. NIH aims to make strides in reducing health outcome disparities through community-driven research efforts (126). The program provides research funding directly to communities who set their own priorities and work in partnership with universities on pressing knowledge gaps in how to improve health outcomes in marginalized communities (127).

Funders have also recognized the need for dedicated expertise (e.g., boundary spanner individuals and boundary organizations) to build strong connectivity between partners and equalize capacity differences (27, 50, 52, 56, 57). While some funders have brought that expertise in-house (see refs. 52, 85, and 87), some have taken the approach of building larger-scale capacity for expert intermediaries or boundary spanners. In the United States, the Rita Allen Foundation, with partners, has made significant investments in the development of experts in connecting research, practice, and policy through the Civic Science Fellows program (128, 129). In the Global South, IDRC has partnered with a range of funders to invest in institutions and networks to strengthen capacity for greater engagement between research, policy, and practice communities. This includes investing in engaged research consortia, as well as consortia coordinators, knowledge brokering organizations, and other boundary organizations who build connective tissue and bridge research and policy (50, 57, 130–132).

Funders have also provided entry platforms for different kinds of partners who are unfamiliar with engaged research to explore participation. Health Research BC, for example, supports REACH BC, a promising on-ramp resource that links researchers and individuals who are interested in participating in health studies as subjects or partners. As partners, volunteers have an opportunity to join in the research where they can help think of new questions, inform research opportunities, and join research advisory committees to share a patient’s point of view (133). The William T. Grant Foundation has also created resources to provide an easy-to-use starting point for researchers and other partners who are interested in engaged research. Their Research-Practice Partnership website provides short guiding tips, recorded webinars, and sample work products (102, 134, 135).

Going forward, we see a need to be willing and able to take a holistic approach to supporting engaged research. This means acknowledging that we need to consider capacity and infrastructure needs for the range of actors who may be involved, including nontraditional research entities (136).

## Embrace A Richer Conception of Impact

Studies of research impact suggest that process impacts, including changes in knowledge, network connectivity, capacities, relationships, attitudes, and problem-framing, may be as important as any discrete policy or practice changes that might eventually occur (137–144). And these are the impacts that practitioners and scholars often find with engaged research efforts. For example, in a recent review of coproduction efforts in sustainability, Norström et al. (6) found that engaged research “processes produce more than just knowledge; they develop capacity, build networks, foster social capital, and implement actions that contribute to sustainability.” Likewise, in education, scholars found that research-practice partnerships, “provide the organizational structure to facilitate sustained collaboration between researchers and practitioners to improve learning opportunities for students” (58).

While these impacts are very different than traditional scientific ones (e.g., high citation counts) or a specific policy or practice change (e.g., a new regulation), they can be measured. Indeed, the William T. Grant Foundation investment in the publicly accessible “use of research evidence” methods repository aggregates promising approaches and provides a collaboration platform for scholars studying the kinds of research impacts described above (115).

We realize embracing a more diverse array of impacts is challenging. Yet, we also know that the process impacts that engaged research offers, e.g., relationship-building and capacity strengthening, are likely to be critical for achieving durable solutions to many societal problems. If we, as funders, intend to improve outcomes over the long term, we have a responsibility to embrace a much richer, more nuanced conception of impact (6, 73).

## Accelerating Change, Carefully

Change is underway. TEFN has grown from twenty or so participants in 2020 to representation from more than 80 different funding organizations at the end of 2023. This growth reflects momentum and interest around the world in efforts to improve research capacity to address complex, societal challenges. For example, the U.S. NSF’s first new directorate in 30 y, the Technology, Innovation and Partnerships directorate, has a focus on engaged research and target budget of $16.3 billion USD over 5 y (145). The Novo Nordisk Foundation in Denmark, with a $114 billion USD endowment in 2023 (146), is investing in engaged research on mobility and global health, where funding proposals must “include close collaboration with local communities, stakeholders, and other relevant actors” (147). UNESCO—a specialized agency of the United Nations—has called on higher education leaders and actors to push for transformation toward transdisciplinary modes of integrating and producing knowledge with diverse societal actors (148, 149). In response to this momentum, TEFN recently launched a university presidents and chancellors council for public impact research to develop a roadmap for supportive infrastructure within universities (150). These examples are just a few of many emerging around the world.

Research funders hold powerful levers to ensure that these approaches are effectively supported and appropriately scaled. They have a responsibility to acknowledge and support the full life cycle of costs in these approaches and to ensure they achieve their intended outcomes. They also have an important role to play in addressing the broader, system-wide barriers grantees and partners face.

As we have described in this perspective, there is much momentum to build on, and a unique opportunity to work across diverse sectors. We are hopeful that if we and other funders embrace new ways of thinking and working, we can accelerate progress toward deep transformations in how to address society's most pressing challenges.

## Data Availability

All study data are included in the main text.
